# Artificial intelligence of imaging and clinical neurological data for predictive, preventive and personalized (P3) medicine for Parkinson Disease: The NeuroArtP3 protocol for a multi-center research study

**DOI:** 10.1371/journal.pone.0300127

**Published:** 2024-03-14

**Authors:** Maria Chiara Malaguti, Lorenzo Gios, Bruno Giometto, Chiara Longo, Marianna Riello, Donatella Ottaviani, Maria Pellegrini, Raffaella Di Giacopo, Davide Donner, Umberto Rozzanigo, Marco Chierici, Monica Moroni, Giuseppe Jurman, Giorgia Bincoletto, Matteo Pardini, Ruggero Bacchin, Flavio Nobili, Francesca Di Biasio, Laura Avanzino, Roberta Marchese, Paola Mandich, Sara Garbarino, Mattia Pagano, Cristina Campi, Michele Piana, Manuela Marenco, Antonio Uccelli, Venet Osmani

**Affiliations:** 1 Azienda Provinciale per i Servizi Sanitari (APSS) di Trento, Trento, Italy; 2 TrentinoSalute4.0 –Competence Center for Digital Health of the Province of Trento, Trento, Italy; 3 Centro Interdipartimentale di Scienze Mediche (CISMed), Facoltà di Medicina e Chirurgia, Università di Trento, Trento, Italy; 4 Casa di Cura Eremo, Arco, Trento, Italy; 5 Department of Medical and Surgical Sciences, Alma Mater Studiorum Università di Bologna, Bologna, Italy; 6 Fondazione Bruno Kessler Research Center, Trento, Italy; 7 Università di Trento, Facoltà di Giurisprudenza, Trento, Italy; 8 IRCCS Ospedale Policlinico San Martino, Genoa, Italy; 9 Department of Neuroscience, Rehabilitation, Maternal and Child Health, University of Genoa, Genoa, Italy; 10 Department of Experimental Medicine, Section of Human Physiology, University of Genoa, Genoa, Italy; 11 DINOGMI Department, University of Genoa, Genoa, Italy; 12 Dipartimento Di Matematica, Università Di Genova, Genoa, Italy; Fondazione Policlinico Universitario Agostino Gemelli IRCCS, ITALY

## Abstract

**Background:**

The burden of Parkinson Disease (PD) represents a key public health issue and it is essential to develop innovative and cost-effective approaches to promote sustainable diagnostic and therapeutic interventions. In this perspective the adoption of a P3 (predictive, preventive and personalized) medicine approach seems to be pivotal. The NeuroArtP3 (NET-2018-12366666) is a four-year multi-site project co-funded by the Italian Ministry of Health, bringing together clinical and computational centers operating in the field of neurology, including PD.

**Objective:**

The core objectives of the project are: i) to harmonize the collection of data across the participating centers, ii) to structure standardized disease-specific datasets and iii) to advance knowledge on disease’s trajectories through machine learning analysis.

**Methods:**

The 4-years study combines two consecutive research components: i) a multi-center retrospective observational phase; ii) a multi-center prospective observational phase. The retrospective phase aims at collecting data of the patients admitted at the participating clinical centers. Whereas the prospective phase aims at collecting the same variables of the retrospective study in newly diagnosed patients who will be enrolled at the same centers.

**Results:**

The participating clinical centers are the Provincial Health Services (APSS) of Trento (Italy) as the center responsible for the PD study and the IRCCS San Martino Hospital of Genoa (Italy) as the promoter center of the NeuroartP3 project. The computational centers responsible for data analysis are the Bruno Kessler Foundation of Trento (Italy) with TrentinoSalute4.0 –Competence Center for Digital Health of the Province of Trento (Italy) and the LISCOMPlab University of Genoa (Italy).

**Conclusions:**

The work behind this observational study protocol shows how it is possible and viable to systematize data collection procedures in order to feed research and to advance the implementation of a P3 approach into the clinical practice through the use of AI models.

## Introduction

Parkinson’s disease (PD) is a degenerative and progressive motor disorder classified as a brain pathology and it is caused by the impairment or death of dopaminergic neurons located in the *substantia nigra* with consequent deprivation of dopamine, the neurotransmitter that allows movement control [1]. In the early stages of the disease, PD patients experience muscle stiffness often associated with slowed movements (bradykinesia), as well as tremor of one or more parts of the body [2]. As the disease progresses, motor symptoms increase in severity and fluctuations in the response to dopaminergic pharmacotherapy typically develop leading to a reduction of the time interval between *off* periods (i.e., when the symptoms of PD are pronounced, and the patient develops a disability because the dopaminergic medication is providing insufficient relief) is reduced. Additional, postural instability i.e., balance impairment affecting the ability to change or maintain postures such as walking or standing might occur, as well as non-motor alteration (behavioral and neuropsychiatric disorder, sleep alteration, cognitive impairment and dysautonomic disorders) [3]. Furthermore, in the long term, drug treatment leads to complications such as motor and non-motor fluctuations and dyskinesias [4–6]. Finally, with disease progression, debilitating complications occur, such as falls or dementia [7].

Despite the fact that pathogenetic mechanisms of PD are not fully known, evidence suggests a multifactorial basis: PD is the result of the complex interaction between physiological aging, genetic predisposition, environmental factors [1]. Family history constitutes a risk factor and in a percentage of cases (5–10%) there are genetic factors both causing or predisposing to PD development, usually with early onset [8]. The use of pesticides among farmers and other work activities involving exposure, in particular, to solvents and heavy metals, appear to be associated with an increased risk. On the other hand, tobacco and coffee smoke appear to be protective factors [9].

The result of the multifactorial basis in PD pathogenesis is that PD is a heterogeneous disease characterized by highly variable motor and non-motor symptoms and patterns of progression. The most recent studies, based on hierarchical cluster analysis, have described a benign subtype characterized by mild motor symptoms (“mild motor-predominant” subtype) and a "diffuse malignant" subtype characterized by the coexistence of severe motor and non-motor symptoms at disease onset [10–12]. Acknowledging these subtypes is important for possible implications for personalized treatment, which is not yet a reality in daily practice [13].

From an epidemiological viewpoint, the burden of PD represents a key public health issue [4]. PD is among the second neurodegenerative diseases in terms of prevalence worldwide, after Alzheimer Disease. It is associated with an increase in mortality and disability, and with considerably high costs, thus implying a substantial burden for patients and their families and caregivers [14] and a heavy socioeconomic load considering the aging population. Therefore, many countries will face a future of unsustainable demands on limited healthcare resources. The prevalence of PD in worldwide population has been described in 2014 in a meta-analysis of 47 epidemiological studies. The meta-analysis estimated a prevalence of PD of 31.5 cases per 10,000 pop (95% CI 11.3–87.3). Data suggest an increase in terms of PD patients up to 9.3 million by 2030 [1, 14].

In Italy the prevalence of Parkinson’s disease is 0.3% in the general population and 1% in subjects over 60 years of age. The disease affects around 250,000 patients, half of whom are working-aged; 6 thousand new cases are registered every year. It is estimated that, due to the growing aging of the general population, the prevalence will double by 2030, and it will become a significant public health problem, considering that the current costs are estimated between 10,000 and 17,000 euros per person [15–17].

Consequently, it is essential to develop innovative and cost-effective approaches to promote sustainable diagnostic and therapeutic interventions. The main goal is to optimize the management of disease, to maximize costs-effectiveness and to tailor delivery of care considering the specific characteristics of the patient, as recommended by WHO in a technical brief titled “Parkinson disease: A public health approach” [1]. The WHO document outlines the global burden, treatment gaps and crucial areas for actions for PD and provides considerations for policies, implementation and research.

In this perspective the adoption of a P3 (predictive, preventive and personalized) medicine approach seems to be pivotal, as recent literature highlights [18–20].

The first necessary step to develop this P3 medicine approach is the standardized collection of data to gain a detailed and harmonized description of the clinical phenotype of PD patients. Correlation of the clinical phenotype with the subsequent progression of PD symptoms, as well as mapping early onset of specific symptoms could play a vital role in improving risk prediction, personalizing treatment and planning appropriate preventive strategies.

To our knowledge, personalized and precision medicine is not yet fully developed. Recent publications reported promising and solid results from a training of Artificial Intelligence (AI)-machine learning algorithms with datasets from approximately 100 patients diagnosed with PD [21–24]. Literature seems to suggest we are at the initial stage of novel approaches in this specific field, including AI-supported approaches [18, 25]. In this scenario, AI-supported tools could allow clinicians to anticipate patients’ needs by estimating clinical trajectories of patients. This could largely improve personalized treatments and appropriate preventive approaches. To develop effective AI-enabled tools, literature and clinical experience suggest that at least three core clinical milestones of the disease should be considered, namely i.e. falls [3, 7], cognitive impairment [21] and disease fluctuations [26, 27]. An AI-roadmap tool to guide personalised treatment for PD could be developed as long as these three milestones are taken into account [28].

In this scenario, the project “Artificial intelligence of imaging and clinical neurological data for predictive, preventive and personalized (P3) medicine” (NeuroArtP3 -NET-2018-12366666) represents a significant step forward a P3 approach to PD supported by AI tools. NeuroArtP3 is a four-year multi-site project, coordinated by IRCCS Ospedale Policlinico San Martino of Genoa and funded by the Italian Ministry of Health within the Italian Regions of the different centers involved. Some centers are involved directly in the design and implementation of the current protocol (IRSSC Ospedale Policlinico San Martino, Azienda Provinciale per i Servizi Sanitari of Trento, Fondazione Bruno Kessler of Trento and Department of Mathematics of the University of Genoa), whilst other centers are participating in the broader implementation of the study targeting diseases other than PD (IRCCS Ospedale San Raffaele of Milan, Istituto Giannina Gaslini of Genoa). The overall project’s core objectives of the project are: i) to harmonize the collection of data across the participating centers, ii) to structure standardized disease-specific datasets and iii) to advance knowledge on diseases trajectories through machine learning analysis.

Through the use of mathematical models, Artificial Intelligence (AI) and machine learning, on a large and common set of data, the project’s goal is to identify patterns of clinical and neuropsychological variables as predictors of the trajectories of PD patients [20]. In particular, the study consists of an initial retrospective phase, aimed at generating preventive, predictive and personalized algorithms, whose value and accuracy will be verified in the subsequent prospective phase, analyzing the correspondence between the mathematical predictions and the actual clinical course of the patients.

The project’s uniqueness lies also in the fact that the list of specific clinical variables adopted to train the AI-algorithms was carefully selected to improve potential usability of the algorithms themselves also in contexts where either basic clinical data is available or data collected with sophisticated technologies are impossible to be generated. The possibility of developing reliable algorithms based on easily accessible clinical data is in line with the guiding principles of the Italian public health system, namely universality, equity, and solidarity [29].

In this paper, authors present the NeurtoArtP3 study protocol, which received approval from the Ethics Committee of the participating centers and which will guide the research implementation.

## Methods

### Study design and participants

The 4-years study combines two consecutive research components: i) a multi-center retrospective observational phase; ii) a multi-center prospective observational phase.

The retrospective phase aims at collecting data of the patients admitted at the participating clinical centers. Whereas the prospective phase aims at collecting the same variables of the retrospective study in newly diagnosed patients who will be enrolled at the same centers. The participating clinical centers are the Provincial Healthcare Trust (Azienda Provinciale per i Servizi Sanitari—APSS) of Trento (Italy) as the center responsible for the PD study and the IRCCS San Martino Hospital of Genoa (Italy) as the promoter center of the NeuroartP3 project. The computational centers responsible for data analysis are the Bruno Kessler Foundation of Trento (Italy) and the LISCOMPlab / department of Mathematics of the University of Genoa (Italy). For the province of Trento the project was designed and implemented with the support and coordination of TrentinoSalute4.0 –Competence Center for Digital Health (Italy).

The overall asset in terms of participating centers and roles is displayed in Table 1.

**Table 1:** Participating centers and roles within the NeuroArtP3 initiative.

	Institute	Department	Role
1	Azienda Provinciale per i Servizi Sanitari, Provincia Autonoma di Trento (Local Healthcare trust)	U.O. Neurologia Ospedale Santa Chiara (Neurology Unit)	Lead clinical center
2	IRCCS Ospedale Policlinico San Martino di Genova	U.O. Neurologia and U.O. Clinica Neurologica (Neurology Unit)	Participating clinical center
3	IRCCS Ospedale Policlinico San Martino di Genova e Università degli Studi di Genova	LisCOMP Lab and Dipartimento di Matematica (Department of Mathematics)	AI modelling
4	Fondazione Bruno Kessler Istituto di Ricerca, Provincia Autonoma di Trento (Bruno Kessler Foundation)	Digital Health and Wellbeing Center	AI modelling

Clinical variables that can predict the course of the disease are identified and selected by the clinical investigators through a harmonization process (as thoroughly described in the following paragraph).

These variables were identified based on two assumptions: i) evidence in the published literature and ii) availability of data within the participating centers.

Within the retrospective cohort database, the centers adopted the following process: i) collecting the data of patients affected by PD; ii) providing longitudinal follow-up from the diagnosis/recruitment (baseline). The follow up is based in annual assessments for a minimum period of 3 years.

The same clinical, biological, imaging, neuropsychological information will be collected in the prospective cohort of patients. For this branch of the study, newly diagnosed patients consecutively referred to the participating Centers are enrolled and periodically evaluated from the clinical onset of the disease (baseline), for a minimum period of 12 months from the date of diagnosis, up to a maximum of 3 years.

Subsequently, the data collected will be analyzed by the computational centers in charge of identifying the AI models and testing their effectiveness in the prospective cohort.

### Inclusion and exclusion criteria

For the retrospective study, the inclusion criteria are defined as follows: being 18 years or older; having a diagnosis of PD (according to UK Brain Bank criteria); attending the participating Centre for at least 3 years prior to recruitment (baseline visit); Hoehn & Yahr (H&Y) staging of 1, 2 or 3 at the baseline visit. Additional details are included in Appendix 2 in S1 File. The Hoehn and Yahr scale is used to describe the symptom progression of Parkinson disease. The scale originally was described in 1967 and included stages 1 through 5. It has since been modified with the addition of stages 1.5 and 2.5 to account for the intermediate course of Parkinson disease. It was designed to be a descriptive staging scale to evaluate both disability and impairment related to clinical disease progression [30, 31].

For the prospective study, the following inclusion criteria are set: being 18 years or older; having a diagnosis of PD; attending the participating Centre in the period August 2020 –June 2023.

Considering the approach adopted in the project and the need for including the largest sample of data available, no specific exclusion criteria have been set. In fact, heterogeneous patients’ profiles will be included in the study with the purpose of testing the computing power and the predictive capacity of the algorithms in the different phases of the disease. For example, we included patients with heterogenous disease duration (less than 5 years, between 5 and 10 years and greater than 10 years).

Within both the retrospective and prospective studies, authors are aware that for newly or recently diagnosed PD patients the diagnosis could turn into atypical parkinsonism [32, 33]. Therefore, particular attention will be paid to monitoring such a potential issue and caution will be taken in data analysis and interpretation.

Patients in the prospective study with different set of comorbidities than those in the retrospective study will not be considered in the analysis. However, the number of such patients is likely to be very low as the choice of comorbidities to be considered was driven by the frequency of occurrence, in addition to the clinical significance.

### Data collection and reference dataset

A set of common data was identified by the participating centers, in order to create a homogeneous dataset with a unified coding system.

Data (including demographic and clinical information) are collected as part of the standard routine practice. This set of routine data was selected to improve possibility of developing and using reliable algorithms based on easily accessible clinical data, in line with the guiding principle of the Italian public health system (universality, equity, and solidarity) [29].

Data from 250 patients will be used at the retrospective phase, whilst a minimum of 50 patients will be recruited for the prospective phase. The numbers of patients were chosen based on the availability of resources as well as existing literature describing prediction of outcomes for patients with Parkinson using machine learning methods [34].

The first set of variables includes pseudo-anonymized demographical patient’s data, where a unique ID is used for linking the different sources of information. A set of different information listed as Appendix (Appendix 1 in S1 File), ranging from year of symptoms’ onset, year of diagnosis and disease duration, plus clinical-anamnestic data. Supplementary data are also considered, when available for a specific patient.

**Fig 1:**
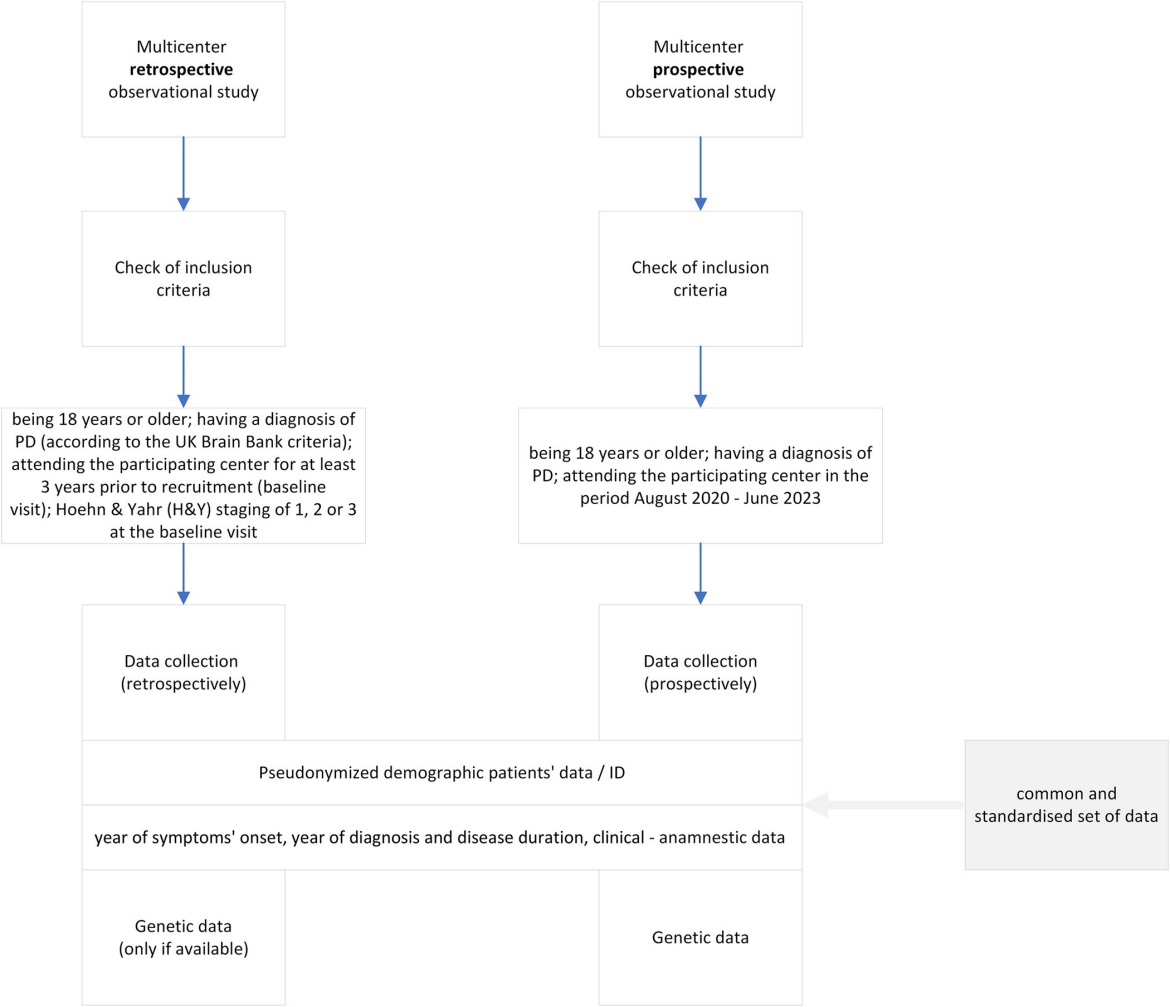
Data collection process.

The data are collected at different timeslots, namely at baseline visit (T0) and at three subsequent follow-ups (at 12, 24 and 36 months, T1-T2-T3). The essential data concern motor, non-motor and pharmacological information, as well as the number of hospitalizations in the last year. The supplementary data includes imaging and genetic information. Fig 1 summarizes the flow of data collection.

The prospective dataset includes the same set of variables collected in the retrospective phase. In addition, for the prospective study only, patients will be asked specific consent for collecting genetic data.

### Data management

REDCap (Research Electronic Data Capture) will be used to host the project database [35] and embedded into the IRCCS Ospedale Policlinico San Martino IT infrastructure. Only staff working on the project is authorized to access. REDCap is designed in line with the European data protection legislation–GDPR.

To guarantee proper standardisation of dataset, the data entry form adopted through REDCap guarantee homogeneous format, labelling process and quality of the collected information. Automated checks in REDCap have been planned to avoid mistyping. Coding methods were chosen by adopting binary/dichotomous variables in the large majority of cases (e.g. hyposmia no = 0, yes = 1), whilst for a limited number of variables a categorical/ordinal value has been identified. This strategy has been adopted to further support low-risk approach in data-entry and to increase homogeneity among the databases. Considering the neuro-imaging pieces of information (e.g. CAT scan), standards of quality of images and files has been agreed based on those provided by the companies. Before data analysis, to further cross-check data comparability a pre-processing and data cleaning phase is foreseen to make sure that the variables from the different centers are homogeneous.

### Ethical and data protection issues

This section summarizes the ethical and data protection issues that have been considered during the research project since the drafting of the study protocol. As anticipated, the project is managed by a consortium of different Italian institutions. The study protocol has been developed through a collaborative approach, involving healthcare professionals, researchers, IT staff and legal experts in order to ensure compliance with clinical and technical standards, and with the ethical and legal rules.

From an ethical point of view, multiple committees have been involved. In particular, the study protocol (NET-2018-12366666-3-PD) has received approval by the following Locals Ethics Committees: Ethics Committee of Azienda Provinciale per i Servizi Sanitari of Trento and of Regione Liguria. To be compliant with the ethical requirements of the Declaration of Helsinki, the guidelines on good clinical practice and the GDPR, the centers asked patients for authorization to participate in the scientific project. The modules for obtaining informed consents describe the study, its goals and characteristics, and explain what participation entails in a plain, clear and transparent way. As regards the legal concerns, high attention has been paid to the governance and management of personal data collected in the project. The adoption of AI for development and training of prediction models implies the use of clinical variables with potential privacy protection issues. This aspect is even more relevant in the case of multi-site projects, where data is collected from different study sites and merged into a unique dataset. This concerted action implies several challenges related to security and privacy, data quality control, data sharing procedures: if not properly addressed, these issues could lead to data concerns, as well as representing a detrimental factor for collaborative research. In the European Union data processing activities should comply with Regulation 679/2016—General Data Protection Regulation (hereinafter: “GDPR”). The centers represent different and autonomous data controllers that collect and process both common and particular categories of data, meaning data concerning health under Art. 9, par. 1, GDPR. However, these subjects concluded a detailed data processing agreement. According to Art. 28 GDPR, specific contracts have also been signed with data processors. The Data Protection Officers (DPOs) of the centers have been involved in the drafting of the documents, including the privacy policies, and they elaborate Data Protection Impact Assessments (DPIA) to map all the data protection risks (Art. 35 GDPR). A data protection by design approach [36] has been adopted to implement organizational and technical measures to safeguard and protect personal data. First of all, to allow proper data sharing and data management of the different sources of data, specific GDPR-compliant software has been adopted. The *ad-hoc* platform is also supporting a specific system to control data access from different environments, in line with security and data protection principles. REDCap (Research Electronic Data Capture) is used to host the project database [35] and embedded into the IRCCS Ospedale Policlinico San Martino IT infrastructure. Only staff working on the project are authorized to access the data. REDCap is specifically designed in line with GDPR requirements: unique keys and strict authorization procedures guarantee confidentiality and integrity of the collected data. Moreover, the implementation of pseudonymization techniques and the separation of databases ensure security of the dataset. Each institution has a unique access to the specific subset of information to be entered into the database. In terms of pseudonymization, patients and related data are linked within the REDCap system with a unique identifier (ID), removing personal identification information like name and surname. The ID code was produced as a progressive alphanumeric code, without even a partial reference to personal identification information that usually are adopted to structure ID codes. The REDCap data entry mask was designed with internal automated checks to detect potential personal data entered by mistake (e.g. entering personal data in the anonymous ID slot), preventing the data entry process to be finalized.

Despite the possibility to avoid the collection of the data subjects’ consent under Art. 110 of the Italian Data Protection Code (D.lgs. 196/2003), the centers decided to ask participants for informed and explicit consent. This choice is in line with a citizen-science perspective, to further guarantee transparency and accountability, as well as to improve patients’ awareness and understanding of the scientific project’s objectives [37, 38].

### Outcomes definition

In line with the literature, three core clinical challenges of the disease have been considered as clinical questions to be addressed through the AI-algorithms, which are reflected as outcomes in the present study. These clinical challenges are identified as falls [3, 7], cognitive impairment [21] and fluctuations [26, 27], reflected into the study outcomes definitions, as follows:

- risk of a fall within the subsequent 3 years, estimated using the patient baseline characteristics and clinical assessment at the initial visit;
- estimating risk of cognitive impairments, specifically mild cognitive impairment (MCI) and dementia within the next 3 years;
- risk of developing symptoms related to motor fluctuations in the next 3 years.

AI algorithms trained in the present study are structured to account for and estimate these three potential risks, considering their pivotal role in determining the development of PD within a patient’s trajectory and their clinical relevance when planning and adapting treatment and care approaches.

### Data analysis and machine learning algorithms

Demographic characteristics and baseline data will be summarized by descriptive statistics using means, standard deviations and 95% confidence intervals for continuous variables, median and inter-quartile ranges for non-normal continuous or ordinal data and percentages for categorical data and will be evaluated for normalcy and homogeneity.

Repeated measures analysis of variance (RMANOVA) and the Kruskal–Wallis test (ANOVA) will be used for normal and non-normal continuous variables respectively to analyze differences across time (baseline vs follows-up). The chi-square test will be used for categorical variables. Statistical significance will be set at p ≤ 0.05.

In terms of machine learning models, we will use tree-based algorithms, such as Random Forest (RF), Support Vector Machines (SVM) and Logistic Regression (LR) (for the baseline), to predict the outcomes of interest. RF is an ensemble of decision trees that provides robust predictive performance using an iterative learning process that sequentially builds many models that correct the deficiencies of the preceding model. We have selected tree-based models as they have shown great predictive performance for structured, tabular data [39], also for clinical applications [40–42]. Furthermore, RF algorithms provide a good compromise between the amount of data available and predictive performance, as is the case with our study. Support Vector Machines are a class of supervised learning methods that can find the optimal separation (hyperplane) between the data points of different classes. SVM can handle high-dimensional data and non-linear relationships between the input data and the outcomes of interest.

We will use LR as a statistical baseline comparator to tree-based models. LR is a statistical method that investigates the relationship between the outcome variable and the input variables and is typically considered as a baseline algorithm in clinical classification tasks.

All the models will be tuned for the best hyperparameters on the internal evaluation cohorts in each study design and outcomes definition. The models’ hyperparameters will be optimized through exhaustive grid-search to maximize the F-1 score metric and then used for the final prospective validation.

### Performance metrics

We will evaluate the predictive performance of the models using area under the receiver operator characteristic curve (AUC) and area under the precision-recall curve (AUPRC), including subgroup analysis [43]. We will also calculate Positive Predictive Values (PPV), Negative Predictive Values (NPV), F-1 scores, and Matthews correlation coefficient (MCC) scores. To increase the transparency of our predictive models, we will use Shapley Additive exPlanations (SHAP) SHAP method deconstructs each prediction into a sum of individual contributions from each variable known as SHAP values. We will use the SHAP values to investigate importance of each input variable with respect to the outcome.

### Dissemination strategy

The Chief Scientific Officer / Principal Investigator is responsible on behalf of the network to produce periodical reports and to ensure that data are reported responsibly and consistently. For the dissemination of results through scientific publications and / or presentation at congresses, conferences and seminars, only aggregated data will be used and in line with the core dissemination principles of safeguarding patients’ rights (e.g. anonymity).

## Discussion

Machine learning approaches are computer-based statistical approaches that can help clinicians in classifying patients according to several variables at the same time and to predict outcomes through AI algorithms. Despite the difficulties of implementing such approaches because of the need for big amounts of homogenous data, the use of machine learning might represent a key cornerstone in developing and applying P3 approaches for complex diseases such as PD [20], as well as personalized medicine in general [18].

This NeuroArtP3 study has the main goal to develop and test AI algorithms, by adopting state-of-the-art methodologies to predict PD trajectories starting from a common set of clinical variables.

The uniqueness of the present initiative lies in different and innovative strands. First, the use of a multi-professional approach embracing different expertise and background in a unique team, including IT specialists, medical doctors, machine learning specialists, privacy and data security experts. This multidisciplinary approach represents a key issue when addressing complex studies, requiring proper balance between scientific rigor, use of innovative methodologies and data security levels in line with the GDPR regulations [36].

A second strength of the study is the concerted harmonization of clinical variables across the different study sites, allowing researchers to use a considerable amount of data for research purposes, in the context of a multi-center study.

A third strand of uniqueness is represented by the adoption of the most advanced methodologies in terms of AI/machine learning. The implementation of these approaches could further enhance the diagnostic performance of the tested algorithms, as well as improve their discriminative capabilities in terms of diagnosis and prognosis and therefore their use to guide and support clinical practice [38].

An additional factor is represented by a list of specific clinical variables adopted to train the AI-algorithms. Data were purposively selected to promote a large use of AI-based algorithms, even in cases where only basic clinical data is available. The possibility of developing reliable algorithms based on easily accessible clinical data reinforce an ethical principle universality and equity in adopting AI solutions in the field of public health [29].

A limited number of studies have collected multi-centre clinical data for Parkinson’s disease, combined with the application of machine learning methods to predict the outcomes of interest [18, 25]. This may be because of the inherent challenges in collecting data from multiple centres, harmonising the collected data as well as harmonising methodologies of data collection. These are the challenges that we have addressed successfully in NeuroArtP3 project

However, few limitations remain.

The first limitation could be linked with the sample size. Despite the number of patients and related data foreseen in the present protocol has been properly identified to address the research questions, an increased sample size could have an impact of the characteristics and performance of the algorithm. Additionally, including a wider spectrum of data could also have an effect on the algorithm characteristics, even if the different sources of data are already foreseen in the present protocol (e.g. laboratory-based data, neuro-imaging information, genetic data).

A second limitation is intrinsically related to the adoption of machine Learning techniques. The black-box effect might significantly represent a limitation in terms of clinical relevance and interpretation of the algorithm, whilst different levels of balancing between increased accuracy and improved explainability could be considered [44]. This will be explored once the preliminary data analysis will be performed.

An additional issue is represented by ethical implications of such algorithms when they are embedded into clinical practice [45]. Even if this goes beyond the objective of this study, the potential impact of an AI tool in the current clinical practice should be further explored. In the current literature there is a debate on the actual (or even potential) effect of these technologies, not to mention the issues related to the wide validation of the AI algorithm as a medical device and the implications in terms of patient/healthcare professional relation and communication [46].

The core expected result of the study is to validate viable and shared procedures of data collection and analysis, that can be embedded into the routine clinical practice of a larger group of clinical centers and hospitals. This would lead to a wide network of institutions promoting a constant process of homogeneous data collection, resulting in the availability of large and standardized datasets to be analyses with innovative AI techniques. Consequently, the generated datasets and algorithms could be applied into standardized protocols of intervention, supporting and guiding clinical practice and improving efficiency in healthcare services. This in view of promoting a sustainable virtuous circle between clinical practice and research, that represent a key action to continuously improve tools and resources for healthcare professionals. The increasing precision of available algorithms to anticipate patients’ needs and trajectories is a key element in putting into action a wide and AI-supported 3P approach.

## Conclusions

AI approaches to study PD require the collection of big amounts of data and concerted efforts are needed to standardize data collection procedures, so that they are applicable also in the context of large multi-site studies. This is even more important considering the absence of reliable biomarkers able to predict PD outcome, and the related need for using existing clinical data, provided that they are properly available in terms of homogeneity and uniformity. The work behind this project shows how it is possible and viable to systematize data collection procedures in order to feed research and to advance the implementation of a P3 approach into the clinical practice though the use of AI models.

In the future, the adoption of a shared approach and same procedures of data collection in multisite studies should became a standard process, allowing the increasing availability a major amount of data and consequently more robust machine learning analyses.

## Supporting information

S1 File (DOCX)
