# Supplementary material for: Artificial intelligence of imaging and clinical neurological data for predictive, preventive and personalized (P3) medicine for Parkinson Disease: The NeuroArtP3 protocol for a multi-center research study
Source: PLoS One. 2024 Mar 14;19(3):e0300127. doi: 10.1371/journal.pone.0300127 (PMC10939244; doi:10.1371/journal.pone.0300127)
Supplement: S1 File — (DOCX) [file pone.0300127.s001.docx]

**APPENDIX 1**

Variables included in PD protocol

| **Patient history** | Patient identification | Patient code  PD symptoms onset  PD diagnosis  Baseline visit data  Disease duration in years | e.g.: 001_PD_RETRO_APSS  Year (yyyy)  Year (yyyy)  Date (dd/mm/yyyy)  Year (yyyy) |
| --- | --- | --- | --- |
|  | Anagraphic data | Date of birth  Sex  Manual dominance  Education  Principal occupation  Abuse of coffee  Smoke  Abuse of alcohol  Toxic exposure  Neurological disease familiarity | Date (dd/mm/yyyy)  Male, female  Right, left  Year (yyyy)  Elementary (e.g.: worker), medium-low (e.g.: not concept employee), medium (e.g.: concept employee), medium-high (e.g.: teacher), high (e.g.: manager)  Yes or not  Yes or not  Yes or not  Yes or not  Parkinson, dementia, multiple sclerosis, amiotrphic lateral sclerosis, brain tumors, others |
|  | Comorbidities | Hypertension  Hypercholesterolemia  Brain injury  Diabetes  Heart disease  Liver disease  Thyroid disease  Non-brain tumors | Yes or not  Yes or not  Yes or not  Yes or not  Yes or not  Yes or not  Yes or not  Yes or not |
| **Essential data (at baseline, 12, 24, 36 months follow-up)** | Patient identification | Patient code  Baseline/12-24-36 fw-up data visit | e.g.: 001_PD_RETRO_APSS  Date (dd/mm/yyyy) |
|  | Motor symptoms | Motor phenotype  H&Y  UPDRS III  Falls  Falls onset  Motor fluctuation  Motor fluctuation onset  Dyskinesia  Dyskinesia onset  Freezing  Freezing onset | Tremor or rigid-akinetic  Score  Score  Yes or not  Before diagnosis, in the first 2 years of illness, from 2 to 5 years, after 5 years of illness  Yes or not  Before diagnosis, in the first 2 years of illness, from 2 to 5 years, after 5 years of illness  Yes or not  Before diagnosis, in the first 2 years of illness, from 2 to 5 years, after 5 years of illness  Yes or not  Before diagnosis, in the first 2 years of illness, from 2 to 5 years, after 5 years of illness |
|  | Cognitive test | MMSE  MoCA | Score  Score |
|  | Non-motor symptoms | Cognitive status  Cognitive status onset  ICD  ICD onset  Dysautonomia  Dysautonomia onset  Depression  Depression onset  Psychosis  Psychosis onset  Hallucination  Hallucination onset  Anxiety  Anxiety onset  Hyposmia  Hyposmia onset  Sleep-wake disorder  Sleep-wake disorder onset | Intact, MCI, dementia  Before diagnosis, in the first 2 years of illness, from 2 to 5 years, after 5 years of illness  Yes or not  Before diagnosis, in the first 2 years of illness, from 2 to 5 years, after 5 years of illness  No, orthostatic hypotension, urinary dysfunction, costipation, other  Before diagnosis, in the first 2 years of illness, from 2 to 5 years, after 5 years of illness  Yes or not  Before diagnosis, in the first 2 years of illness, from 2 to 5 years, after 5 years of illness  Yes or not  Before diagnosis, in the first 2 years of illness, from 2 to 5 years, after 5 years of illness  Yes or not  Before diagnosis, in the first 2 years of illness, from 2 to 5 years, after 5 years of illness  Yes or not  Before diagnosis, in the first 2 years of illness, from 2 to 5 years, after 5 years of illness  Yes or not  Before diagnosis, in the first 2 years of illness, from 2 to 5 years, after 5 years of illness  No, RBD, insomnia  Before diagnosis, in the first 2 years of illness, from 2 to 5 years, after 5 years of illness |
|  | Pharmacological data | Levodopa  Duodopa  Dopamine agonist  IMAO  ICOMT  Anti-dementia | Year first prescription  Assumption or not  Posology  Controlled release  Stalevo  Year first prescription  Assumption or not  Posology  Year first prescription  Not, pramipexole, ropinerolo, rotigotine  Posology  Year first prescription  Not, selegiline, rosaniline, safinamide  Posology  Year first prescription  Not, entacapone, tolcapone, opicapone  Posology  Yes or not |
|  | DBS | Intervention year  Intervention type | Year (yyyy)  STN, PPN, GPI, other |
|  | Hospitalization | Reason | Not, PD-related cause, PD-non related causes |
|  | MRI imaging | T1 3D  FLAIR T2 | DICOM  DICOM |
| **Supplementary data at baseline** | PET imaging | FDG-PET  DATSCAN  MIBG | DICOM  DICOM  DICOM |
|  | Quantitative EEG | -- | DICOM |
|  | PSG | -- | DICOM |
|  | Smell test | -- | Score |
|  | Genetic test | -- | Yes or not |

**APPENDIX 2**

Diagnostic criteria for idiopathic Parkinson disease developed by the United Kingdom Parkinson’s Disease Society Brain Bank

| Diagnostic criteria for idiopathic Parkinson disease developed by the United Kingdom Parkinson’s Disease Society Brain Bank |
| --- |
| 1. Diagnosis of parkinsonian syndrome:  Bradykinesia and ≥1 of:  1) Muscular rigidity  2) 4-6 Hz rest tremor  3) Postural instability not caused by primary visual, vestibular, cerebellar, or proprioceptive dysfunction |
| 2. Exclusion criteria for idiopathic Parkinson disease:  1) History of repeated strokes with stepwise progression of parkinsonian features  2) History of repeated head injury  3) History of definite encephalitis  4) Oculogyric crises (episodes of involuntary gazing, caused by tonic spasms of extraocular muscles)  5) Neuroleptic treatment at onset of symptoms  6) ≥1 affected relative  7) Sustained remission  8) Strictly unilateral features after 3 years  9) Supranuclear gaze palsy  10) Cerebellar signs  11) Early severe autonomic involvement  12) Early severe dementia with disturbances of memory, language, or praxis (ability to perform complex intentional movements)  13) Babinski sign  14) Presence of a cerebral tumor or communicating hydrocephalus on computed tomography  15) Negative response to high doses of levodopa (if malabsorption excluded)  16) Exposure to MPTP |
| 3. Supportive prospective positive criteria for idiopathic Parkinson disease (≥3 required for diagnosis of definite Parkinson disease):  1) Unilateral onset  2) Resting tremor present  3) Progressive disorder  4) Persistent asymmetry affecting the side of onset most  5) Excellent response to levodopa (>70%)  6) Severe levodopa-induced chorea  7) Levodopa response for ≥5 years  8) Clinical course of ≥10 years (typical course of Parkinson disease) |
| Adapted from [J Neurol Neurosurg Psychiatry. 1988 Jun;51(6):745-52](https://pubmed.ncbi.nlm.nih.gov/2841426/). |
